# Understanding positional cues in salamander limb regeneration: implications for optimizing cell-based regenerative therapies

**DOI:** 10.1242/dmm.013359

**Published:** 2014-06

**Authors:** Catherine D. McCusker, David M. Gardiner

**Affiliations:** Francisco J. Ayala School of Biological Sciences, Department of Developmental and Cell Biology, University of California Irvine, CA 92602, USA.

**Keywords:** Integration, Limb regeneration, Positional information, Regenerative medicine, Stem cell

## Abstract

Regenerative medicine has reached the point where we are performing clinical trials with stem-cell-derived cell populations in an effort to treat numerous human pathologies. However, many of these efforts have been challenged by the inability of the engrafted populations to properly integrate into the host environment to make a functional biological unit. It is apparent that we must understand the basic biology of tissue integration in order to apply these principles to the development of regenerative therapies in humans. Studying tissue integration in model organisms, where the process of integration between the newly regenerated tissues and the ‘old’ existing structures can be observed and manipulated, can provide valuable insights. Embryonic and adult cells have a memory of their original position, and this positional information can modify surrounding tissues and drive the formation of new structures. In this Review, we discuss the positional interactions that control the ability of grafted cells to integrate into existing tissues during the process of salamander limb regeneration, and discuss how these insights could explain the integration defects observed in current cell-based regenerative therapies. Additionally, we describe potential molecular tools that can be used to manipulate the positional information in grafted cell populations, and to promote the communication of positional cues in the host environment to facilitate the integration of engrafted cells. Lastly, we explain how studying positional information in current cell-based therapies and in regenerating limbs could provide key insights to improve the integration of cell-based regenerative therapies in the future.

## Introduction

The adult human body is composed of many different types of cells arranged according to specific instructions to form tissues and organs that interconnect harmoniously and function as a complete biological unit. This is a result of events that occur during embryogenesis, which coordinate the patterning and differentiation of cells and gradually build up the complexity of the structures throughout our bodies until we reach adulthood. This developmental process is also recapitulated during regeneration in organisms that can regenerate complicated body structures, such as limbs. However, one key difference is that the cells of the regenerating system arise from and are connected to adult tissues that have already completed patterning and differentiation. Thus, regeneration requires not only the development of new pattern and the differentiation of cells to fit into this pattern, but also for the new pattern to align perfectly with the existing pattern, and for the new cells to differentiate and seamlessly interconnect with the cells in the existing tissues. We define this process as ‘integration’ and, without it, the completed structure will not function as a cohesive biological unit.

The long-term vision of regenerative therapies is to replace or repair complicated biological structures that have been damaged through traumatic injury or disease. This will require the generation of specified cell types, which will have to be organized and integrated into the existing structures. For example, the heart is composed of many cell types, including cardiomyocytes, cardiac pacemaker cells, Purkinje fibers, smooth muscle cells, fibroblasts, epicardium and endothelial cells, that are organized and interconnected according to a specific framework to maintain the structural, mechanical and electrical properties of the heart. Injury to the heart, for instance by myocardial infarction, results in the death of a variety of these cell types, which must then be replaced, organized and interconnected in the correct way in order to reestablish the normal function. Moreover, failure to repair and integrate the new heart tissue into the existing heart tissue puts stress on the heart as a whole, increasing the risk of heart failure. Eyes are also a good example of complicated structures that are composed of a variety of specialized cell types. Cell-based regenerative therapies designed for repairing damaged eyes need to ensure that the engrafted cells are not only organized and integrated in a specific way to regain the function of the eye, but are also wired properly into the neural circuitry so that our brains can process the images in a way that we can understand. Similarly, damage to regions of the body such as the face or limbs require the replacement and organization of many different types of tissues, and efforts to repair these structures should not only focus on regaining the function of the damaged body part, but also to repair the structure in a way that is esthetically comparable to the original structure so as to maintain life quality. These examples illustrate that, in addition to knowing how to generate the ‘building blocks’ (i.e. specialized cell types) of the damaged organ, we must also know how to organize and integrate these new cells into the existing structures of the body. Understanding the basic biology of tissue integration could therefore help us to improve cell-based regenerative therapies.

Many studies have focused on how to differentiate cells into specific somatic lineages so that they can be used as the building blocks to repair the damaged tissues (reviewed in Tabar and Studer, 2014). However, the role of positional information in the integration of grafted cell populations into the damaged host environment is less well studied. As an embryo develops, each cell learns its location relative to the different axes of the body plan. This cellular property, known as positional information, is epigenetically encoded, and has been shown to persist in some cells throughout adulthood in both salamanders and humans (French et al., 1976; Bryant et al., 1981; Rinn et al., 2006). In particular, the mesenchymal compartments of adult tissues are known to be rich in cells with positional memory, such as fibroblasts (Bryant et al., 2002; Chang et al., 2002; Nacu et al., 2013). The positional information in embryonic and adult cells provides instructive cues that can modify the molecular signature and behavior of the surrounding tissues and drive the formation of new structures (French et al., 1976; Rinn et al., 2008). Thus, cells with positional information can have powerful morphogenic activity, which, if unregulated, could lead to dangerous consequences such as uncontrolled cell growth during cancer development (Cheng et al., 2005; Ruiz i Altaba et al., 2007).

With the advent of cell-based therapies, researchers are now attempting to place cells into a complicated three-dimensional host environment that contains cells with positional information (Fisher and Mauck, 2013). However, little is known about the positional interactions that occur between the grafted cells and the host environment and how this can affect the behavior of these cells. To what extent does positional information play a role in the integration of grafted cells into the pattern of the host environment? What problems could positional information impose on the utility of cell-based therapies? Understanding positional information and how it controls the behavior and integration of cells is likely to provide insight into how to improve the efficacy of cell-based therapies. In this Review, we focus on the role of positional information in eliciting different cellular behaviors during the process of salamander limb regeneration. Salamanders are among the few vertebrates that are capable of regenerating complicated biological structures as adults (Nye et al., 2003). Thus, they are excellent models to understand the role of positional information in adult tissues, which in turn could guide the optimization of cell-based therapies.

## The integration of new tissue into existing tissue depends on the positional information in both tissues

**Fig. 1.**
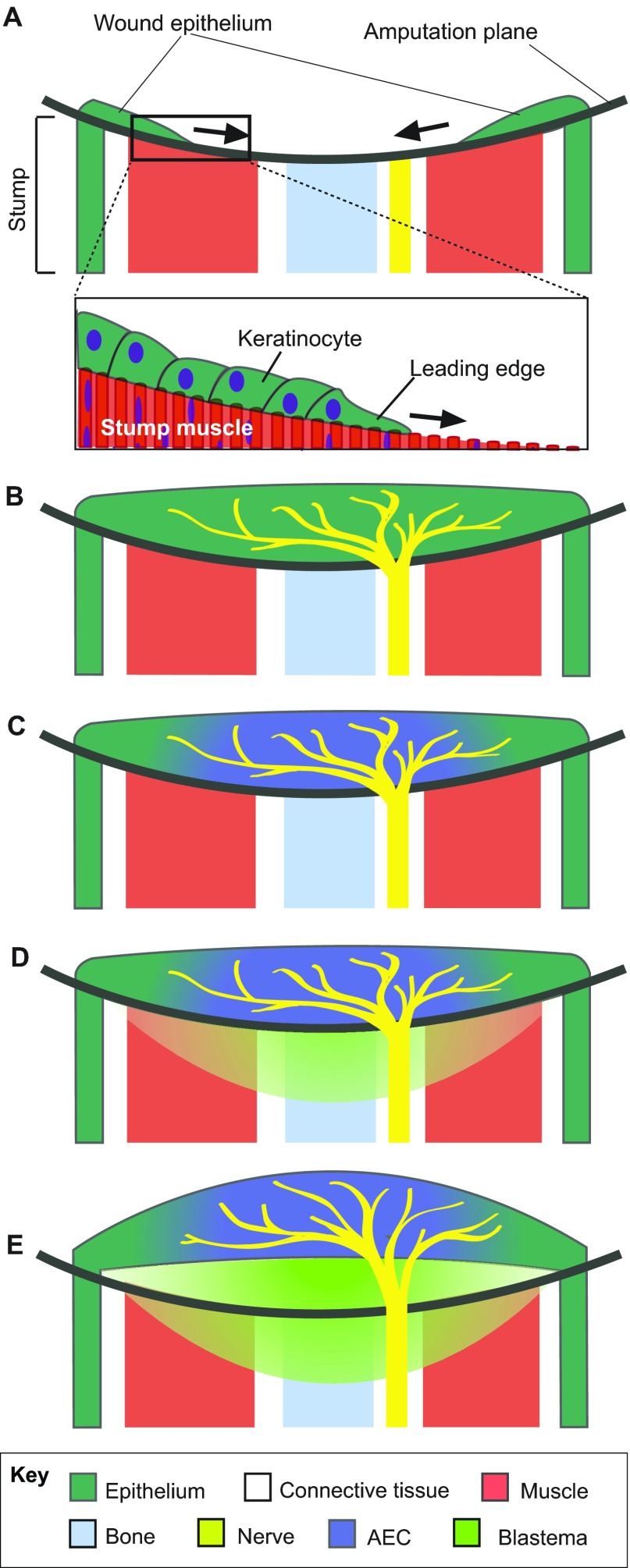
**The establishment of a regeneration-competent environment during salamander limb regeneration.** The diagram is a longitudinal view of the salamander limb during the early stages of blastema formation, showing the establishment of a regeneration-competent environment (A–D) and early blastema formation when positional interactions between cells are initiated (E). (A) Immediately after the limb is amputated, basal keratinocytes of the surrounding epidermis migrate across the injured stump tissues to form a wound epithelium. (B) Regenerating nerve fibers grow into the wound epithelium. (C) The nerve–wound-epithelium interactions generate the apical epithelial cap (AEC) signaling center. (D) Signaling from the AEC stimulates the dedifferentiation of stump tissues to generate blastema cells. (E) Early blastema cells accumulate below the wound epithelium, where they communicate information about their original position in the limb.

Salamander limb regeneration requires the formation of a group of regeneration-competent limb progenitor cells called the blastema at the severed end of the limb to regenerate the missing structures (Fig. 1). One of the crucial components required in order for a blastema to form is the generation of a wound epithelium that covers the site of injury. The wound epithelium becomes innervated from the regenerating ends of an underlying nerve (Singer, 1974). Innervation induces the wound epithelium to become a specialized signaling center, called the apical epithelial cap (AEC), that produces a number of growth factors and morphogens, which act as chemoattractants and mitogens to attract and expand the blastema cell population (Endo et al., 2004; Ghosh et al., 2008; Mullen et al., 1996). Connective tissue cells from different positions around the circumference of the limb stump migrate into the wound site, interact and communicate information about their position of origin, which establishes that there is a positional disparity (i.e. something is missing). Once cells with different positional information interact, cell proliferation is stimulated and cells with the intermediate positional identities are generated until the pattern of the missing structure is complete (Bryant et al., 1981; French et al., 1976). Because the blastema cells differentiate into the different tissues of the limb in accordance with this pattern, the regenerated tissues seamlessly integrate with the existing (old) limb structures.

**Fig. 2.**
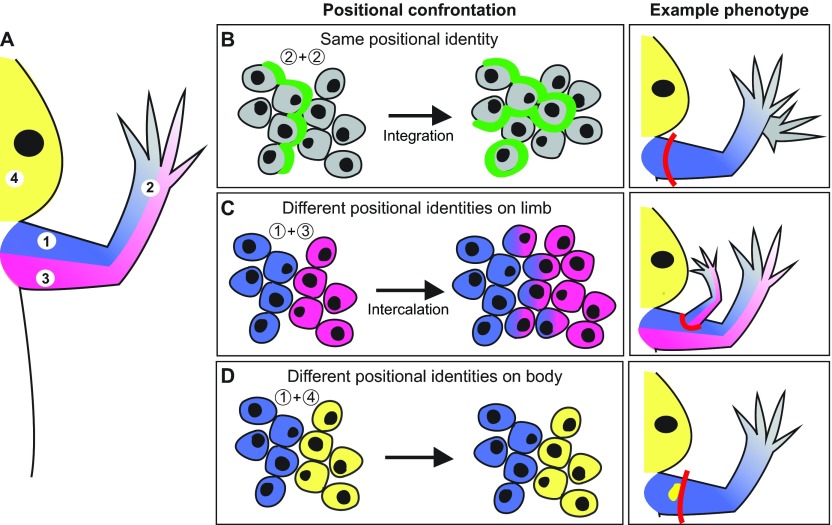
**Behaviors elicited by different positional confrontations.** (A) Diagram of the right half of a salamander, indicating the regions from which cells were obtained for positional confrontation experiments (1, anterior limb; 2, distal limb; 3, posterior limb; 4, head). (B–D; left) Positional confrontation. The diagrams represent what happens when cells generated from the host blastema are juxtaposed (confronted) with graft cells in a regeneration-competent environment. The diagram on the left shows the groups of cells that are confronted; the diagram to the right of this shows the final cell positioning. (B–D; right) Example phenotype. The red line indicates the amputation plane where the host blastema forms. Note that the posterior limb is shown in pink only when anterior and posterior interactions are being tested. (B) The confrontation of cells from the distal limb (gray) with other cells from the distal limb (gray) leads to integration between these cells. The green line indicates the hypothetical boundary between the cell confrontations of the grafted and host cells, which integrate (shown by merging of cells to the left and right of the green line) because they have the same positional information. An experimental example of this is when a medium-bud blastema from a distal amputation is grafted to a proximally located blastema. The grafted distal blastema cells integrate with the host cells in the distal region of the proximal blastema, resulting in a regenerated limb with two hands (right) (as described in Crawford and Stocum, 1988). (C) The interaction of anterior limb cells (blue) with posterior limb cells (pink) leads to integration by intercalating cells with the intermediate positional identities (blue/pink). An example of this phenotype is when posterior cells are grafted into an anterior wound site and a limb field with the intermediate values is intercalated, resulting in the formation of an ectopic limb (right) (as described in Endo et al., 2004). (D) The confrontation of limb cells (blue) with cells from the newt head (yellow) does not result in integration. An experimental example of this phenotype is when head cells are grafted into a limb (as described in Satoh et al., 2007; Tank, 1987). The confrontation does not result in the intercalation of cells with positional information between the head and limb, and does not result in the formation of ectopic structures in the regenerated limb.

Studies on regenerating salamander limbs have provided key insights as to how the positional interactions between tissues affect their integration with a host environment. Below, we provide a few examples from grafting studies on regenerating limbs that illustrate specific behaviors elicited when cells with varying degrees of positional disparity interact with each other (Fig. 2).

### (1) Cells with the same positional information will integrate

Perhaps one of the best examples that illustrates this rule is from an experiment in which blastemas from different positions along the proximal-distal axis of the limb were grafted to a proximally located (i.e. upper arm) blastema (Crawford and Stocum, 1988). Over time, each of the grafted blastemas integrated into the region of the host blastema that corresponded to their original proximal-distal location (i.e. the region of the host blastema with the same proximal-distal positional information) (Fig. 2B). For example, a blastema graft from a proximal site integrated with the proximal region of the host blastema, whereas a graft from a distal site integrated with the cells with distal positional information in the host blastema. This ability of blastema tissues to integrate with other tissues with similar positional information is thought to be mediated by cell adhesion molecules, because blastema cells from different regions on the limb will segregate from each other if mixed together, a property known as selective adhesion (Nardi and Stocum, 1984).

### (2) Cells from different regions of the limb must resolve their positional discontinuity in order to integrate

When cells from one position on the limb are grafted into a new position on the limb, growth is stimulated to generate cells with the intermediate positional information. For example, when limb tissue from the posterior (i.e. back) region of the limb is grafted into a wound site in the anterior region (i.e. front) of the limb, the positional discontinuity is resolved by generating a new limb field, and results in the formation of an ectopic limb (Fig. 2C) (Endo et al., 2004). This process of generating cells with intermediate positional information that resolve a positional discontinuity between cells is called ‘intercalation’ (Bryant et al., 1981; French et al., 1976). Thus, limb cells with different positional information integrate by stimulating intercalation that bridges the gap in positional information.

### (3) Cells with positional information from different regions of the body do not integrate

In contrast to above, juxtaposing (or confronting) cells from different positions in the body does not always result in an intercalary response. For example, grafts of dermal fibroblasts from the head or flank region into a regenerating limb do not participate in the regenerative response and are passively displaced by limb cells as the new limb forms (Fig. 2D) (Satoh et al., 2007; Tank, 1987). Thus, the positional discontinuity between the grafted cells from the head region and the cells of the limb host environment is not resolved through an intercalary response, and thus grafted cells do not integrate into the host tissues. This observation suggests that grafts of cells with positional information from some regions of the body are incapable of integrating with limb tissues. However, more studies that involve grafting tissues from different regions of the body into the limb will be required to test how universal this property is.

It is important to note that cells need to be in a permissive environment to communicate their positional identity (Fig. 1). In all of the above examples, the tissue grafts interacted with host cells that had been induced to become regeneration-permissive as a result of signals from the nerve and the wound epithelium. As discussed above, a regeneration-competent environment is produced by the interaction of nerves with the wound epithelium, which induces the dedifferentiation of mature limb cells that contribute to the blastema (Endo et al., 2004; Satoh et al., 2010). In the absence of dedifferentiation (blastema formation), when mature limb tissues with differing positional information are grafted, there is no intercalary response (Carlson, 1975). Similarly, if signaling from the nerve is removed, the blastema cells prematurely differentiate (McCusker and Gardiner, 2013) and the intercalary response ceases (Mullen et al., 1996; Singer, 1946; Suzuki et al., 2005). Thus, it seems that dedifferentiation is necessary for cells to become able to communicate and respond to positional cues, and, thus, integration is also dependent on dedifferentiation.

## The role of positional information in the efficacy of cell-based regenerative therapies

Given the importance of positional information in constructing and integrating new tissues during an endogenous regenerative response, it is likely that it will also play a role in these processes in cell-based regenerative therapies. Many induced pluripotent stem cell (iPSC) lines that are currently being developed for cell-based therapies are derived from connective tissue cells (fibroblasts), which are the cells that have positional information. Fibroblasts have been a major source of parent cells for iPSC lines because they are an easily obtained source of cells to establish patient-specific cell lines (Shtrichman et al., 2013). However, fibroblasts also express a position-specific molecular fingerprint (Rinn et al., 2006), and are capable of communicating this information and altering the expression of region-specific molecules in surrounding cells (Muneoka et al., 1986; Rinn et al., 2008).

Despite much having been learned about how to reprogram fibroblasts in terms of generating specific differentiated phenotypes, very little is known about the positional memory in these cells. Hox genes, which are essential for patterning of the early embryo, are associated with positional memory in adult cells, and are differentially expressed in fibroblasts from different regions of the human body (Rinn et al., 2006; Rinn et al., 2008). Region-specific expression of Hox genes is stabilized through epigenetic modifications in fibroblasts throughout the connective tissues in the body (Rinn et al., 2007). Hox genes are differentially expressed in iPSCs derived from different somatic sources (Chen et al., 2013), suggesting that a region-specific Hox code might be retained in these iPSC populations. Consistent with this idea, it was observed that the methylation pattern of *HOXD8* was maintained in a number of iPSC lines even after culturing for an extended period of time (Kim et al., 2011), which has been shown to promote the epigenetic reprogramming of iPSCs in terms of somatic identity (Guenther et al., 2010).

**Fig. 3.**
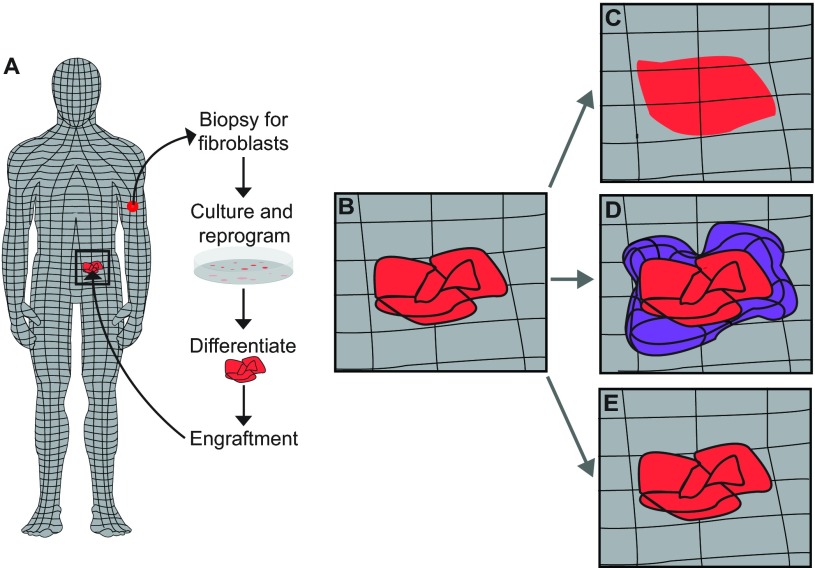
**Potential outcomes from grafting cells with positional information into a human host environment.** (A) Connective tissue cells have information about their position on the adult body (represented as a grid). Cell-based therapies that use populations of cells that were derived from parental cells from one location on the body (e.g. limb), to graft into a different region on the body (e.g. abdominal cavity) are potentially confronting cells with differing positional information. (B) The alignment of positional information (black lines) in the host (grey) and grafted (red) tissues is minimal immediately after engraftment. Over time, multiple integration phenotypes could emerge (C–E). (C) One possible outcome is that the positional information in the grafted cells aligns with that of the host environment, the tissues integrate perfectly (as described in Fig. 2B) and function is restored. (D) Another possibility is that, in an attempt to resolve the positional discontinuity via intercalation, cells with intermediate positional information are generated (purple), resulting in ectopic tissue growth (as described in Fig. 2C) and failure to restore function in the damaged host tissues. (E) It is also possible that the grafted cells fail to align their positional information with the host cells and the tissues fail to integrate (as described in Fig. 2D), and thus have diminished (or no) functionality.

Based on what we know about confronting cells with different positional information in a regenerated limb (Fig. 2), if fibroblast-derived iPSCs that retain positional memory are grafted into a host site that possesses different positional information, they could either fail to integrate or could induce an intercalary response that results in the growth and formation of aberrant structures, such as during teratoma formation (Fig. 3). It would be informative to compare the epigenetic profiles on Hox genes of iPSCs derived from parent cells of the same tissue origin but different location within that tissue to determine whether the residual Hox code differs depending on the specific position from which the parent cells were obtained. Additionally, it would be interesting to determine whether grafted cells that were derived from parent populations that were located in a region with either similar or different positional information as the host environment have different potentials to integrate or induce ectopic growth. Lastly, experiments that test whether ectopic induction of a Hox code in grafted cell populations to match the Hox code of the host site promotes integration and, conversely, whether altering the Hox code in cells that were generated from iPSCs derived from parent populations from the same location as the host site induces defective integration phenotypes (i.e. failure to integrate or formation of ectopic growth). These, and other, future studies will help us understand the positional interactions between donor and host cells to determine the extent to which they play a role in these integration phenotypes.

If fibroblast-derived iPSC lines do retain positional information, the good news is that their positional information could be manipulated to be compatible with the information in the host site, which would promote integration. Recent studies have shown that the positional information of early blastema cells of connective tissue origin is plastic, and that these cells can be reprogrammed if grafted to a position on the limb that is different from their position of origin (McCusker and Gardiner, 2013). Although the specific molecular mechanisms that induce and maintain this plastic state are yet to be discovered, nerve signaling is required (McCusker and Gardiner, 2013). Understanding the basic biology behind positional plasticity will be important for improving the integration of therapies using cell populations that retain positional information.

Another way of modifying the positional information of engrafted cells is through the activation of the retinoic acid (RA) signaling pathway. Treatment of a regenerating salamander limb with exogenous RA results in the reprogramming of the positional information of blastema (dedifferentiated) cells to a more proximal identity, whereas the information in the differentiated limb stump tissue is unaffected (McCusker et al., 2014; Maden, 1982; Niazi et al., 1985). For example, if a blastema from a mid-forearm (i.e. mid-radius and ulna) amputation is treated with RA, an entire new limb is regenerated, rather than just the missing distal lower arm and hand (Maden, 1982). The effect of RA on patterning of the regenerate is dose-dependent such that higher levels of ectopic RA for longer periods of time results in more severe patterning phenotypes than lower doses over shorter time periods (Maden, 1983; Tickle et al., 1985). RA could be a useful tool to alter the positional program in tissue grafts to better match the positional information in the host site in cell-based therapies; however, the expression of the proper RA receptor (RAR) isoforms in the engrafted cells will be important for positional reprogramming. In support of this, although a number of RAR isoforms are expressed in the salamander limb blastema, only one of these forms, δ_2_, is responsible for the reprogramming phenotype described above (Pecorino et al., 1996). Additionally, although the effect that ectopic RA has on reprogramming the positional information in the limb regenerate has been well documented since the 1980s, nothing is known about the mechanisms driving this activity. Understanding where activated RARs interact with the DNA in reprogrammed blastema cells will not only bring us closer to determining which genes play a role in programming positional information, but also how positional information is reprogrammed.

## Cells without positional information respond to positional cues from connective tissue cells in order to integrate

Thus far, we have discussed the behaviors elicited when cells with positional information (i.e. cells of connective tissue origin) interact and how this affects the integration of these cells. However, not all cells have positional information; rather, they respond to the positional cues from neighboring connective tissue cells. One way to determine whether a particular cell type has positional information or not is by testing whether it can induce the formation of a new pattern when confronted with cells with different positional information in a regeneration-competent environment. For example, grafts that include connective tissues such as the dermis will induce the formation of an ectopic limb pattern if grafted to a different region on the salamander limb (Rollman-Dinsmore and Bryant, 1982; Tank, 1981). On the other hand, grafted skeletal muscle cells, Schwann cells and epidermal cells do not induce the formation of ectopic pattern, but rather merge into the pattern of the new host environment (Kragl et al., 2009; Nacu et al., 2013). Thus, connective tissue cells use their positional memory to regenerate the new pattern, and other tissues such as muscle, Schwann cells and epidermis respond, and merge into this pattern (see Bryant et al., 2002). This behavior also seems to hold true for mammals, because altering the positional information in mouse dermal fibroblasts results in re-regionalization of neighboring epidermal cells *in vivo* (Rinn et al., 2008).

It has been suggested that connective tissue cells use Wnt proteins to communicate positional information to the surrounding cells. In cultured mouse cells, Wnt5A [a ‘distal’ Wnt (expressed in distally located tissues and is required for distal limb development)] can rescue the expression of distal markers in epidermal cells that have been co-cultured with dermal fibroblasts with depleted HoxA13 (a ‘distal’ Hox protein) (Rinn et al., 2008). *Wnt5A* is also present in the salamander limb regenerate, and ectopic expression of Wnt5A in the blastema interferes with the formation of a complete limb (Ghosh et al., 2008), suggesting that Wnt5A and potentially other Wnts could play a role in communicating positional information in the regenerating limb. Cell-cell interactions are also thought to play an important role in the communication of positional information from signaling cells to responding cells in developing systems (French et al., 1976; Bryant et al., 1981; Bryant and Gardiner, 1992). A growing body of evidence in invertebrate and vertebrate models supports the idea that molecules that provide positional cues are transported through cell protrusions that extend from the signaling cell to the responding cells (Roy et al., 2014; Sanders et al., 2013). However, there is much to learn about the mechanisms and molecules that connective tissue cells use to communicate positional information to neighboring cells in adult tissues.

A number of cell-based therapies involve engrafting cell populations that do not themselves carry positional information, but they nevertheless will require positional cues from the connective tissue cells in the host environment in order to integrate properly. One example that illustrates this challenge is the use of embryonic stem cell (ESC)-derived neural progenitors as a source of cells for the regeneration of neurons. ESC-derived neural progenitors survive when grafted into a variety of regions in the mammalian brain and form synaptic connections with host cells at the site of engraftment, but they do not reestablish the appropriate patterns of innervation necessary to restore normal function (Weick et al., 2011; Wernig et al., 2004). Thus, the engrafted cells fail to respond to the position-specific molecular map of the surrounding neural tissues (Wernig et al., 2004) that helps guide axons to make the proper connections in the brain rather than aberrant connections that send signals to the wrong target cells (Colak et al., 2013). Miswired neuronal circuits are thought to have a causal role in a number of human neuropsychiatric diseases, including depression, social anxiety disorder and schizophrenia (Furman et al., 2011; Klumpp et al., 2013; Mitchell, 2011). For example, aberrant neural projections from the cuneus to the corpus callosum have been associated with abnormal social behavior in individuals with Williams syndrome (Marenco et al., 2007). Thus, it is important that the engrafted cells form the appropriate connections in the neural network. Although much effort has been concentrated on differentiating different sources of neural progenitor cells into the various neuronal derivatives (Jongkamonwiwat and Noisa, 2013), little is known about how to guide these cells to form the correct connections into the neural circuitry and thus integrate into the host in a functionally appropriate manner.

It is possible that ESC-derived neural progenitor cells form aberrant connections to cells in the host because positional cues are not communicated in that environment. If this is the case, these cell-based therapies could be improved by treating the host environment so that positional information can be communicated to the grafted cells. In regenerating limbs, treatment with specific signaling molecules can generate an environment in which cells with positional information can interact. Combined administration of the growth factors FGF2, FGF8 and GDF5 (also known as BMP14) induces a regeneration-permissive environment by mimicking nerve signaling during endogenous regeneration (Makanae et al., 2013). Thus, the application of these molecules could be used to facilitate the communication of positional cues from the connective tissue cells in the host environment to the grafted cells.

## Conclusions and future perspectives

Positional information is a fundamental property of embryonic and adult cells that controls how cells interact and respond to each other, and thus is likely to be an important factor in integrating grafted cells into a host environment in cell-based regenerative therapies. In this Review, we have considered current therapies where either the presence or absence of positional information could cause defective integration phenotypes, and have discussed molecular tools that could be used to manipulate the positional information in cells, as well as their ability to communicate this positional information in future cell-based therapies. Based on how cells with positional information interact during limb regeneration, we hypothesize that human cell grafts that retain positional information could either fail to merge into the pattern of the host tissues, or induce an ectopic growth response. Because RA reprograms positional information in regenerating limbs, this could be a useful tool to reprogram the positional information in the grafted cells to better match the host environment and improve the integration of these tissues. At the very least, studies using ectopic RA in regenerating salamander limbs show that positional information can be reprogrammed in adult cells, although positional reprogramming has yet to be shown in adult mammalian systems. Additionally, we speculate that generating an environment in which positional information can be communicated from connective tissue cells in the human host environment to grafted cells that do not have positional information (such as nerves) could be important for making the appropriate connections between these tissues to restore normal function. This environment can be induced through the application of specific growth factors (FGF2, FGF8 and GDF5) in salamanders, and these or other growth factors could be used to generate an environment that promotes the communication of positional information.

At the present time, little is known about the positional information in engrafted cell populations in mammalian systems, and nothing is known about how positional interactions between the grafted and host populations affects the integration of these tissues. Thus, there is a crucial need for more studies that focus on positional information in cell-based regenerative therapies in mammalian systems. Future studies in regenerating limbs will also be required to understand how positional interactions work and are modified in adult tissues. We have much to learn about how positional interactions can be harnessed to promote the integration of engrafted cell populations with the host environment for cell-based regenerative therapies.
